# The last two transmembrane helices in the APC-type FurE transporter act as an intramolecular chaperone essential for concentrative ER-exit

**DOI:** 10.15698/mic2024.01.811

**Published:** 2024-01-05

**Authors:** Yiannis Pyrris, Georgia F. Papadaki, Emmanuel Mikros, George Diallinas

**Affiliations:** 1 Department of Biology, National and Kapodistrian University of Athens, Panepistimioupolis, Athens, 15784, Greece.; 2 Department of Pharmacy, National and Kapodistrian University of Athens, Panepistimioupolis, Athens, 15771, Greece.; 3 Institute of Molecular Biology and Biotechnology, Foundation for Research and Technology, Heraklion, 70013, Greece.

**Keywords:** Aspergillus nidulans, transporter, membrane traffic, NCS1, sorting, ER exit

## Abstract

FurE is a H^+^ symporter specific for the cellular uptake of uric acid, allantoin, uracil, and toxic nucleobase analogues in the fungus *Aspergillus* nidulans. Being member of the NCS1 protein family, FurE is structurally related to the APC-superfamily of transporters. APC-type transporters are characterised by a 5+5 inverted repeat fold made of ten transmembrane segments (TMS1-10) and function through the rocking-bundle mechanism. Most APC-type transporters possess two extra C-terminal TMS segments (TMS11-12), the function of which remains elusive. Here we present a systematic mutational analysis of TMS11-12 of FurE and show that two specific aromatic residues in TMS12, Trp473 and Tyr484, are essential for ER-exit and trafficking to the plasma membrane (PM). Molecular modeling shows that Trp473 and Tyr484 might be essential through dynamic interactions with residues in TMS2 (Leu91), TMS3 (Phe111), TMS10 (Val404, Asp406) and other aromatic residues in TMS12. Genetic analysis confirms the essential role of Phe111, Asp406 and TMS12 aromatic residues in FurE ER-exit. We further show that co-expression of FurE-Y484F or FurE-W473A with wild-type FurE leads to a dominant negative phenotype, compatible with the concept that FurE molecules oligomerize or partition in specific microdomains to achieve concentrative ER-exit and traffic to the PM. Importantly, truncated FurE versions lacking TMS11-12 are unable to reproduce a negative effect on the trafficking of co-expressed wild-type FurE. Overall, we show that TMS11-12 acts as an intramolecular chaperone for proper FurE folding, which seems to provide a structural code for FurE partitioning in ER-exit sites.

## INTRODUCTION

Transporters are transmembrane proteins that mediate selective uptake or export of solutes, metabolites, ions and drugs across the plasma membrane (PM) or other organellar membranes. Secondary active transporters of the PM couple the transport of substrates against their concentration gradients with the transport of other solutes down their concentration gradients. All PM transporters, despite their structural, functional and evolutionary differences, operate via an alternating access model [1], where substrates bind on transporters from one side of the PM and elicit conformational changes, which lead to the opening of the transporter on the other side of the membrane, thus enabling release of substrates [2–4]. Mechanistic variations of the alternating access model, known as the rocker-switch [5], the rocking-bundle [6] or the elevator-type transporters [7], reflect major structural differences among specific transporters. Notably, however, in all three mechanistic models, transporters undergo significant structural changes from an outward-facing to an inward-facing conformation, and vice versa, via a series of substrate-occluded structures [4].

The Amino Acid-Polyamine-Organocation (APC) superfamily is one of the largest and highly ubiquitous families of secondary transporters [8, 9], including well-studied transporters of biomedical interests, as for example neurotransmitter transporters DAT (dopamine) or SERT (serotonin), or transporters mediating the uptake of nucleobase-related drugs (e.g. 5-fluorouracil or 5-flurocytosine). APC transporters are characterized by a 5+5 α-helical inverted repeat (known as the 5HIRT or LeuT fold) formed by ten continuous transmembrane segments (TMS1-10). The ten TMSs are arranged in two discrete domains, the so-called ‘hash’/scaffold domain (TMS3, TMS4, TMS8, TMS9) and the ‘bundle’/core domain (TMS1, TMS2, TMS6, TMS7). In this arrangement, TMS5 and TMS10 function as dynamic gates controlling access to and release of substrates/cations from a central binding site. The substrate binding site in APCs is made by residues located in TMS3, TMS6, TMS8 and TMS10, at the interface of the hash and bundle domains. Based on this fold, all APC transporters function via variations of the rocking-bundle model, where the outward-facing to inward-facing conformational change occurs by the relative motion between the bundle or hash motifs, which underlies substrate accessibility and release [10–14]. It has been suggested that substrate binding in the outward-facing conformation is assisted by the simultaneous binding of a positive charge ion (Na^+^ or H^+^), which elicits the conformational change of the protein towards the inward-facing conformation. Recent high-resolution structures further showed that water molecules shape and stabilize the substrate-binding site and affect the functioning of gates in the bacterial AdiC, APC-type, transporter [15]. Notably also, the cytosolic N- and C-terminal regions of several APC transporters have been shown to be involved in intramolecular interactions that are critical for function, substrate specificity or transporter turnover [16].

Most APC transporters possess two ‘extra’ TMSs at their C-terminal part (e.g. TMS11 and TMS12), the role of which does not seem to be directly related to substrate transport catalysis. Although the majority of APC structures have been resolved as monomeric transporters, in some cases, it has been proposed that these two extra C-terminal TMSs might be critical for APC oligomerization. For example, the AdiC transporter has been crystallized as a stable dimer where the homodimer interface is formed by non-polar amino acids from TMS11 and TMS12 [15]. In this case, however, the role of APC oligomerization remains dubious as each monomer seems to be a self-contained transporter [15, 17]. LeuT has also been crystalized as a dimer via TMS9 and TMS12, and possibly TMS11 [18, 19], but to our knowledge there is no information whether dimerization is necessary for transport activity. There is also evidence, via co-immunoprecipitation [20], crosslinking studies [21] and FRET experiments [22, 23], that DAT and SERT transporters oligomerize. Similar results supporting oligomerization have been reported for rGAT1 and glycine transporters [24, 25]. However, in the aforementioned cases, only in SERT, TMS11 and TMS12 have been shown to be implicated in oligomerization *in vivo* [26].

The APC superfamily includes the Nucleobase Cation Symporter 1 (NCS1) group, of which several fungal and plant transporters have been extensively studied at the genetic and functional level [27–34]. In particular, work performed in *Aspergillus nidulans*, a filamentous fungus that has developed to be a model system to study transporters [35–37], has unveiled important knowledge on the regulation of expression, subcellular trafficking, turnover, transport kinetics and substrate specificity of NCS1 transporters [27–31]. All *A. nidulans* NCS1s function as H^+^ symporters selective for uracil, cytosine, allantoin, uridine, thiamine or nicotinamide riboside and secondarily for uric acid and xanthine. Previous studies have modeled *A. nidulans* NCS1s using the homologous prokaryotic Mhp1 benzyl-hydantoin/Na^+^ transporter [10] as a structural template, and assessed structure-function relationships via extensive mutational analyses. These studies have identified the substrate binding site and substrate translocation trajectory, and revealed important roles of the cytosolic N-and C-terminal segments in regulating endocytic turnover, transport kinetics and, surprisingly, substrate specificity of NCS1 transporters [27–31]. Noticeably, all characterized functional mutations in NCS1 map in specific TMSs of the 5+5 inverted repeat fold or in the cytosolic terminal regions of these transporters.

Here, we systematically investigate the role of TMS11 and TMS12 in the most extensively studied NCS1 transporter of *A. nidulans*, namely the allantoin-uracil-uric acid/H^+^ FurE symporter. We show that two specific aromatic residues in TMS12 are essential for ER-exit and traffic to the PM, apparently via structural interactions with specific residues in the core domain (TMS1-10) that catalyzes transport. We subsequently provide genetic evidence that TMS11-12 is essential for oligomerization or/and partitioning of FurE in specific membrane microdomains to achieve ER-exit and traffic to the PM.

## RESULTS

### Most residues in TMS11 and TMS12 of FurE are not critical for PM localization and have a moderate role in transport

Previous studies concerning NCS1 transporters failed to show a functional role of residues of the last two TMSs (TMS11-12) in transport kinetics or substrate specificity [28,31]. In FurE specifically, where we have employed several unbiased genetic screens to select functional mutants, we have never obtained any mutation located in TMS11 or TMS12 affecting FurE function. In line with this, the reported distinct crystal structures (outward-facing, substrate-occluded or inward-facing) of the Mhp1 bacterial NCS1 homologue strongly suggested that TMS11 and TMS12 do not participate in transport catalysis [13, 38–42].

**Figure 1:**
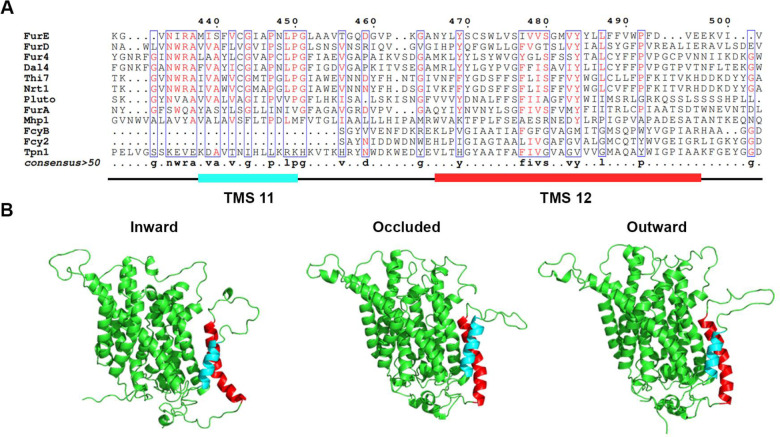
FIGURE 1: TMS11-TMS12 form a distinct structural entity topologically distinct from the LeuT-fold. **(A)** Structural models of FurE in three conformations have been produced through homology modelling using the crystallographically resolved structures of its bacterial homolog Mhp1 as a template. TMS11 and TMS12 are highlighted in cyan and red, respectively. PyMOL 2.5 was used for structure depiction. For details on model construction see methods. **(B)** Multiple sequence alignments of a segment of representative NCS1 family transporters from fungi, plants and bacteria, including TMS11 and TMS12. Positions with >50% identity are presented in boxes. For uniport IDs and taxonomy see Supplementary Table S7.

In order to investigate whether the last two TMSs of FurE play any role in transport activity, substrate specificity, subcellular trafficking or turnover, we constructed strains expressing triple alanine (Ala) substitutions of residues predicted to form the helices of TMS11 and TMS12 (**Figure 1A**; for details see Materials and methods). The choice of Ala substitution is based on the logic that Ala residues conserve the hydrophobic nature of these transmembrane segments but replace specific amino acid side chains that might be important for function. The predicted structures of FurE, shown in **Figure 1B**, have been constructed via homology modeling with Mhp1. By comparing the outward-facing or occluded structures to the inward-facing conformation of FurE, what becomes immediately apparent is a significant distancing of TMS11-12 from the main body of the transporter (TMS1-10), also associated with a tilt of the cytosolic-facing half of TMS12, which is now exposed towards the lipid bilayer (marked in red In **Figure 1**). The significance of this observation becomes apparent later.

**Figure 2:**
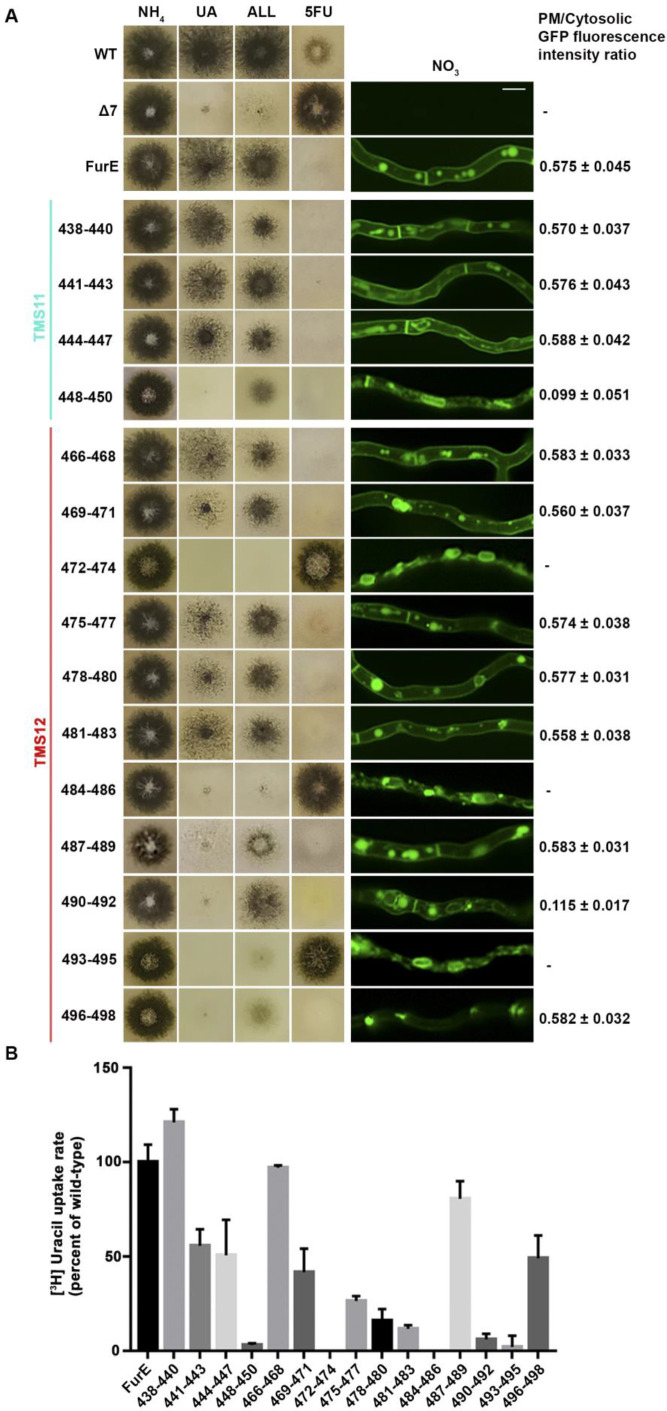
FIGURE 2: Functional analysis of triple Ala mutations in TMS11 and TMS12. (A) Left panel: Growth tests of control strains and strains expressing GFP-tagged mutant FurE versions with triple alanine substitutions that cover the entire length of TMS11 and TMS12. WT is a standard *A. nidulans* wild-type strain. Δ7 is the negative control strain lacking all major nucleobase transporter and not expressing FurE. FurE is the positive control strain, which is a Δ7 strain functionally expressing FurE via the *gpdA* promoter. All mutant strains are isogenic to the positive control strain, also express-ing FurE versions from the *gpdA* promoter. Mutants are named by the number of residues substituted by Ala. Growth tests were performed on minimal media (MM) supplemented with ammonium (NH_4_), uric acid (UA), allantoin (ALL) as nitrogen sources or on MM containing NO_3_ and the toxic analogue 5-fluorouracil (5FU). **Middle panel**: Epifluorescence microscopy images of the same control and mutant strains. Scale bar is 5 μM. Right panel: PM/cytosol GFP fluorescence intensity ratios. 95% confidence intervals are pre-sented. For details see methods. **(B)** Ra-diolabeled uracil uptake rates, expressed as % of wt FurE rate, of strains expressing triple alanine substituted versions. For details see Materials and Methods.

**Figure 2A** shows growth tests relevant to FurE function of all triple Ala mutants and control strains. As also shown previously [30, 31], wild-type (wt) FurE expressed via the *gpdA* promoter in a genetic background lacking all other major nucleobase transporters (e.g., Δ7) confers growth on allantoin or uric acid as sole nitrogen sources and leads to sensitivity to 5-fluorouracil (5FU). The ‘empty’ Δ7 isοgenic strain lacking FurE expression (negative control), cannot grow on allantoin or uric acid and is resistant to 5FU. Notice that uracil, although it is also a FurE substrate, is not used as a N source in *A. nidulans*. Most of triple mutations did not significantly affect FurE-dependent growth on allantoin or sensitivity to 5FU, resembling the growth phenotype of the strain expressing the wt FurE. Some mutants, mostly those concerning TMS12, showed reduced growth on uric acid, which is characteristic of lower transport capacity of FurE. Notably, two triple Ala replacements in TMS12, those affecting residues 472–474 and 484–486 led to total loss of FurE function. Substitutions of residues 448–450, which mark the end of TMS11, but also of 487–490 and 490–492 led to partial loss-of-function of the FurE transporter, evident through the growth defects exhibited by the respective strains on uric acid. Finally, the very last triple Ala mutant (496–498), which corresponds to the beginning of the cytosolic C-tail, also led to a drastic reduction in FurE function (reduced growth on allantoin, significant sensitivity to 5FU and loss of growth on uric acid).

To investigate whether the loss or reduction of FurE function in specific mutants is due to problematic translocation to the PM, reduced protein stability, or defective transport activity per se, we took advantage of the fact the all FurE alleles made were functionally fused in their C-terminus to a GFP epitope. **Figure 2A** (middle panel) shows the *in vivo* subcellular localization of wt and mutant versions of FurE, as followed by widefield epifluorescence microscopy. The two TMS12 mutations leading to apparently total FurE loss-of-function (472–474 and 484–486) led to retention of the transporter in the ER (notice the prominent rings, typical of nuclear ER in fungi; see also Supplementary Figure S1). Mutation 493–495, which led to nearly total loss of FurE function also showed prominent ER-retention. Also, substitutions 448–450 and 490–92 led to partial ER-retention of FurE, rationalizing the growth phenotypes of the corresponding strains. In all other cases, FurE mutant versions label the PM, septa and vacuoles, similar to a correctly folded wt FurE transporter [30, 31]. Quantification of the ratio of PM-associated to cytosolic FurE-GFP fluorescence shows that most functional mutants give a similar result with that obtained with wt FurE, suggesting that the levels of FurE expression is in these strains is comparable (**Figure 2A**, right panel and Supplementary Figure S2). Only mutations concerning residues 448–450 and 490–492 showed ∼5-fold reduced quantity of PM-associated FurE relative to the wt control, concomitant with partial ER-retention of the transporter.

We performed direct uptake assays of all mutants made to test whether the mutant versions of FurE conserve transport capacity for radiolabeled uracil (radiolabeled allantoin is not available and radiolabeled uric acid is unstable). **Figure 2B** shows that most mutants conserve detectable transport rates, albeit often at a reduced degree. Transport rates measured ranged from those similar to wt FurE (i.e., close to 100%, in 438–440, 466–468 and 487–489) to moderate (∼44–55%, in 441–443, 444–447, 469–471 and 496–498), low (∼15–30%, in 475–477, 478–480, 481–483) or extremely low (just detectable, <10%, in 448–450, 490–492 and 493–495). As might have been expected, the 472–474 and 484–486 Ala triple mutants, which showed ER-retention of FurE, did not exhibit detectable FurE transport capacity. Thus, uptake assays are in good agreement with growth tests and microscopy, which showed that most mutations, except those retained in the ER, could confer normal or reduced FurE-mediated growth on allantoin or uric acid and were sensitive to 5FU. Notice that for most transporters of nitrogen sources (e.g., amino acids, purines, nitrate, urea, etc.) studied in our laboratory, analogous mutations allowing transport rates >10%, of the respective wt transporter are sufficient to confer detectable growth, while mutations allowing transport rates >25% of wt can grow normally.

In summary, our results showed that most of the residues in TMS11 and TMS12 are not essential for proper folding and localization of FurE to the PM and are not absolutely essential for transport. The exception concerns three amino acid triplets, namely 472–474, 484–486 and to a lower degree 493–495, which seem to contain sequence-specific information essential for proper ER-exit and trafficking to the PM, and thus for function. Among these mutations, 472–474 (Ser-Trp-Leu) and 484-486 (Tyr-Tyr-Leu) include conserved aromatic residues, Trp473 and Tyr484, respectively. Especially Tyr484 is absolutely conserved in all Fur-like transporters, replaced by aliphatic hydrophobic acids (Met or Val) in the homologous Fcy-like subgroup of NCS1 transporters (see **Figure 1A**). Noticeably also, Tyr484 is one helix turn downstream from the tilt-point (Gly481) where the TMS12 ‘breaks’ during the transition from outward- or occluded to the inward-facing conformation (see **Figure 1B**). Thus, we decided to investigate the role of these two aromatic residues in more detail.

### Tyr484 is irreplaceable for proper ER-exit of FurE possibly due to interactions with specific residues in TMS3, TMS10 and TMS12

**Figure 3:**
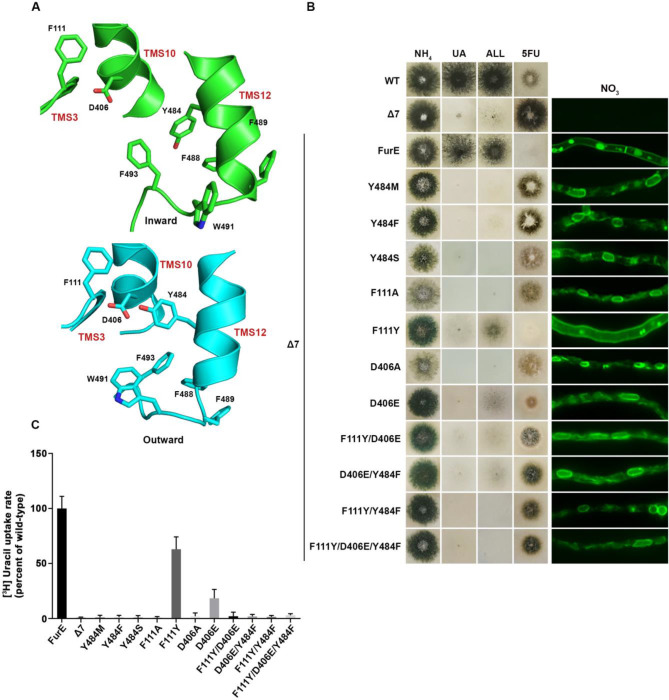
FIGURE 3: Y484 is irreplaceable for proper folding and ER-exit, mediating critical interactions between the LeuT-fold and TMS12. **(A)** Structural models of FurE in the inward- and outward-facing conformations highlighting important interactions in the Tyr484 neighbourhood. Notice the tilt of TMS12 between the two conformations that directs Tyr484 closer to Asp406 (TMS10) and Phe111 (TMS3) in the outward conformation. PyMOL 2.5 was used for structure depiction (see also Supplementary figure S2). **(B)** Growth tests and epifluorescence microscopy of control strains and strains expressing GFP-tagged FurE mutations in Tyr484 (TMS12), Phe111 (TMS3) and Asp406 (TMS10). Growth tests were performed as described in figure 2A. Scale bar for microscope images is 5 μM. **(C)** Radiolabeled uracil uptake rates, expressed as % of wt FurE rate, of strains expressing FurE mutant versions. For details see Materials and Methods.

We constructed single Phe, Ser or Met replacements of Tyr484, the only well conserved residue of the triplet 484–486. The rationale for constructing and analyzing the Y484M is based on the observation that Met is present in the Fcy-type subgroup of NCS1 transporters, which exhibits distinct and non-overlapping substrate specificity (see **Figure 1A**). All three mutations led to inability for growth on allantoin and uric acid and resistance to 5FU (**Figure 3B**, left panel). This was shown to be due to ER retention, similar to what was found with the triple Ala replacement of residues 484–486 (**Figure 3B**, right panel). In line with growth tests and microscopy, direct uptake studies of radiolabeled uracil showed that Phe, Ser or Met replacements of Tyr484 led to FurE loss of function (**Figure 3C**). Thus, neither the presence of hydroxyl group (Ser) nor of an aromatic ring (Phe) could functionally replace Tyr484. In conclusion, Tyr484 is shown to be irreplaceable for the proper function of FurE, apparently due its essential role in ER-exit and translocation of the transporter to the PM.

Defective ER-exit could be due to improper intramolecular folding, defective interaction with annular lipids, or due to abortive interactions with Sec24, the main membrane cargo receptor, or specific ER chaperones [43]. To investigate these possibilities, we tried to select genetic revertants suppressing the lack of growth on allantoin of strains expressing FurE-Y484F or FurE-Y484S. We failed to obtain any, after repeated trials. This might suggest that Tyr484 is involved in complex interactions, these being intramolecular or with lipids or other proteins.

We tried to identify, by modeling, the location of Tyr484 in respect to other domains of the transporter or the lipid bilayer. In the outward and occluded conformation Tyr484 faces the interior of FurE and seems to be at interacting distances with Phe111 located in the beginning of TMS3, and with Asp406 located towards the last turn of TMS10 (**Figure 3A**). More specifically, Tyr484 is predicted to interact via a H-bond with Asp406 and through pi-pi stacking with Phe111. Interestingly, in the inward-facing conformation, as a consequence of a tilt of half of the TMS12 helix, the side chain of Tyr484 although still oriented to Asp406, also gains direct contacts with the lipid bilayer. Consequently, the interaction with Asp406 and Phe111 is weakened (**Figure 3A** and Supplementary Figure S3). This local conformational change is probably related to the final step of the transport cycle. Noticeably also, Tyr484 is also in close distance, especially in the inward conformation, with a series of aromatic acids in the end of TMS12, namely Tyr485, Phe488, Phe489, Trp491 and Phe493, which come into contact with the lipid bilayer (Supplementary Figure S3).

The above findings suggested that the essentiality of Tyr484 might be associated to dynamic interactions with specific residues in TMS3 and TMS10, but also with downstream aromatic residues at the end TMS12, close to the cytoplasmic interphase with the lipid bilayer. If so, we thought that mutations in the interacting amino acids of Ty484, namely residues Phe111 and Asp406, but also Phe488, Phe489, Trp491 and Phe493, might lead to functional defects similar to those of mutation in Tyr484. Noticeably, Phe111 and Asp406 are nearly absolutely conserved in NCS1 transporters, while the other aromatic residues are only partially conserved in eukaryotic homologues.

To further investigate the idea of a network of functional interactions of Tyr484 with Phe111 and Asp406 we constructed and analysed strains carrying the following FurE mutations: F111A, F111Y, D406A, D406E, F111Y/D406E, F111Y/Y484F, D406E/Y484F and F111Y/D406E/Y484F. Notice that the double mutation F111Y/Y484F inverts the topology of the aromatic acids involved, while some of the other mutations involve very conservative changes (e.g., F111Y or D406E). Nearly all of these amino acid substitutions scored as loss-of-function mutations associated to retention of FurE in the ER, very similar to Y484F (**Figure 3B**). An exception was only F111Y, one of the most conservative changes, which did not affect trafficking to the PM, but still led to a small defect in activity, reflected as reduced growth on allantoin and no growth on uric acid. Direct uptake assays were in excellent agreement with growth tests and microscopy (**Figure 3C**). The similarity of defects in ER-exit and FurE activity caused by mutations in Tyr484, Phe111 and Asp406 was in good agreement with the network of interactions of these residues, as proposed via structural modeling. Finally, notice that Ala mutations in amino acids triplets in TMS12 that include Trp491 and Phe493 also showed partial (490-492) or significant (493-495) ER retention, resembling the effect of replacing Tyr484 (see **Figure 2A**). Altogether, it seems that a network of interactions between residues in TMS12 and TMS3 or/and TMS10 might indeed be necessary for proper folding of FurE, and thus essential for its proper exit of FurE and traffic to the PM.

### An aromatic residue at position 473 is necessary for FurE ER-exit

**Figure 4:**
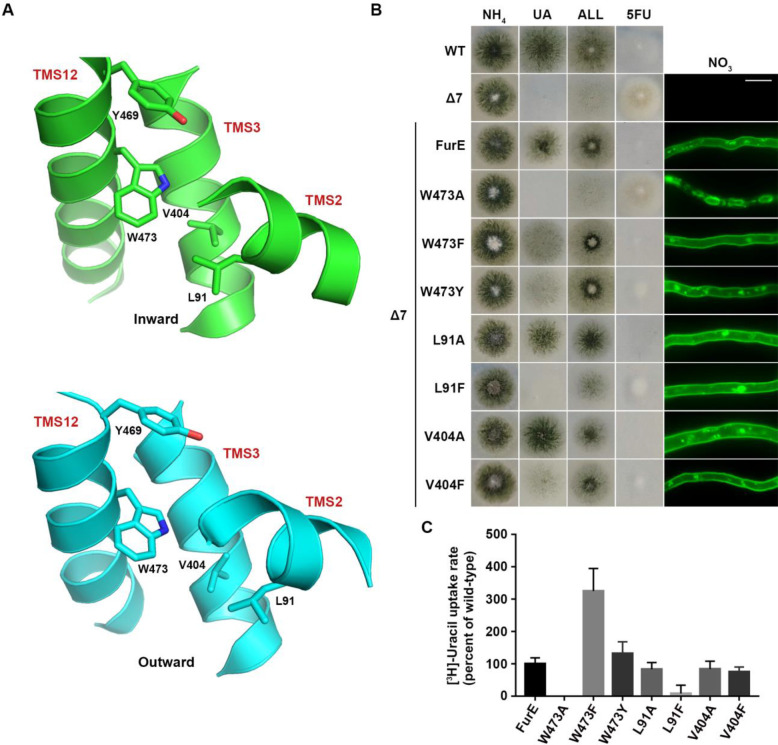
FIGURE 4: The aromatic character of W473 is critical for FurE folding. **(A)** Structural models of FurE in the inward- and outward-facing conformations depicting putative hydrophobic interactions of Trp473 (TMS12) with Val404, (TMS10), Leu91 (TMS2) and Tyr469 (TMS12). PyMOL 2.5 was used for structure presentation. **(B)** Growth tests and epifluorescence microscopy of controls and strains expressing GFP-tagged FurE mutations in Trp473, Leu91 and Val404. Scale bar for microscopy is 5 μM. **(C)** Radiolabeled uracil uptake rates, expressed as % of wt FurE rate, of strains expressing FurE mutant versions. For details see Materials and Methods.

We also constructed and analysed W473A, W473F and W473Y substitutions of the well-conserved Trp473 (**Figure 4A;** see also **Figure 1A**). W473A conferred a FurE-dependent growth defect, which similarly to the Tyr484 mutations was shown to be related to ER-retention (**Figure 4B**). In contrast, both W473F and W473Y substitutions were functional, conferring growth on allantoin or uric acid and sensitivity to 5FU, and also showing proper localization of FurE to the PM (**Figure 4B**). In fact, W473F seems to enhance the presence of FurE protein in the PM, as the respective mutant not only shows increased PM associated fluorescence, but also the fraction detected in cytosolic foci originating from endocytosis (vacuoles and endosomes) is relatively reduced when compared to the image of wt FurE. Direct uptake measurements of radiolabeled uracil were in agreement with growth tests and microscopy, as W473A showed no transport activity, while W473F and W473Y exhibited increased (3-fold) or similar to wt transport rates, respectively (see **Figure 4C**). Thus, it seems that the presence of an aromatic residue at 473, not necessarily a Trp, is sufficient for proper ER-exit and FurE function. Notice that an aromatic residue is conserved in all Fur-like homologues.

Molecular Dynamics (MD) were employed to identify putative interactive residues or whether Trp473 faces the membrane lipids. This showed that Trp473 is exactly in the middle of the lipid bilayer plane and might interact with Ile87, Pro88 and principally Leu91 in TMS2, Ala400 and Val404 in TMS10, and less so with Tyr469 and Ile477 in TMS12 (**Figure 4A**). The sites of residues corresponding to Leu91, Ala400, Val404 and Ile477 are conserved as aliphatic residues in other FurE homologues transporters, while Tyr469 is conserved as Tyr or Phe in all Fur proteins. The predicted interactions with the aforementioned residues appear rather stable. Trp473 has also contacts with lipid chains as the indole moiety is oriented in several structures towards the lipid-protein interface. In the outward and inward structure there are lipid chains parallel to the aromatic ring, but in the inward structure TMS12 appears to be slightly tilted exposing Trp473 further to lipid contacts (Supplementary Figure S4).

Based on the above observations we mutated Leu91 and Val404 into Ala or Phe residues. The functional analysis of mutations L91A, L91F, V404A and V404F showed that none of them affects FurE localization to the PM. However, the Phe substitutions led to reduced growth on allantoin and uric acid and increased resistance to 5FU, whereas the Ala substituted mutants behaved as wt FurE in growth tests (**Figure 4B**). Uracil uptake assays confirmed that Ala substitutions did not affect FurE transport capacity, while Phe substitution led to differentially reduced FurE activity (**Figure 4C**). Substitution Y469A, concerning another residue possibly interacting with Trp473, is included in the already analyzed triple Ala mutation of residues 469-471, which showed no FurE defect. Overall, unlike the case of Tyr484, we could not identify putative intramolecular partners of Trp473 critical for FurE trafficking to the PM. Thus, the molecular basis that underlies the essentiality of an aromatic residue at position 473 for proper FurE folding and ER-exit remains elusive, although it might be related to dynamic interactions of TMS12 with TMS2 and/or TMS10, and probably with the membrane lipids too, as judged by MD.

### Evidence for oligomerization or concentrative partitioning of FurE molecules at ER-exit sites

Previous reports have suggested that APC transporters might dimerize or oligomerize via their TMS11 and TMS12 domains (see Introduction). To address this issue in FurE and try to better understand the role of these segments in ER-exit and transport activity, we co-expressed ER-retained mutant versions of FurE with wt FurE. Co-expression was achieved by co-transformation of the Δ7 strain with plasmids carrying different FurE alleles expressed via the *gpdA* promoter (for details see Materials and methods).

**Figure 5:**
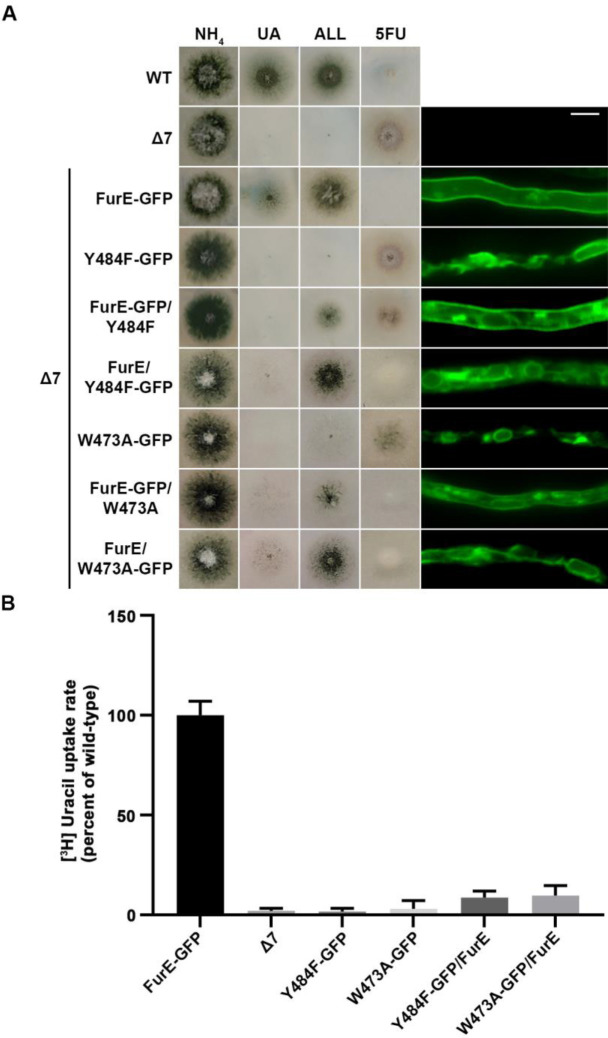
FIGURE 5: ER-retained mutants affect wild-type FurE localization, resulting in reduced transport activity. **(A)** Growth tests and epifluorescence microscopy of controls and strains co-expressing wild-type FurE with ER-retained FurE mutant versions. GFP-tagging is either on the wt FurE or in the ER-retained mutant. Notice the appearance of fluorescent signal associated with ER membranes (mostly as perinuclear rings) in the case were wt FurE-GFP is co-expressed with untagged Y484F or W473A. Scale bar corresponds to 5μM. **(B)** Radiolabeled uracil uptake rates, expressed as % of wt FurE rate, of strains expressing FurE mutant versions. For details see Materials and Methods.

**Figure 5** shows results obtained with selected transformants co-expressing FurE-Y484F or FurE-W473A with wt FurE. In both cases, we analyzed transformants where the GFP epitope is tagged in either the ER-retained mutant or the wt FurE. This allowed us to address the effect on subcellular localization of the ER-retained version on wt FurE and vice versa. As already discussed, both FurE-Y484F or FurE-W473A cannot confer growth on allantoin and uric acid and cannot accumulate the toxic analogue 5FU, while the strain expressing wt FurE grows on allantoin and uric acid and is sensitive to 5FU. Strains co-expressing ER-retained FurE-Y484F or FurE-W473A with wt FurE showed intermediate growth phenotypes (**Figure 5, left panel**). That is, they exhibited reduced growth on allantoin, no growth on uric acid, and intermediate resistance to 5FU. Epifluorescence microscopy of the same strains rationalized the growth phenotypes obtained (**Figure 5, right panel**). The strains co-expressing the ER-retained versions and wt FurE tagged with GFP showed partial ER retention of wt FurE-GFP (see panels with FurE-GFP/FurE-Y484F and FurE-GFP/FurE-W473A). Strains co-expressing the ER-retained versions tagged with GFP and untagged wt FurE showed no PM labeling, similar to the original ER-retained mutants. In agreement with the growth tests, direct uptake assays with radiolabeled uracil confirmed that FurE transport rates are significantly reduced in the strains co-expressing ER-retained FurE-Y484F and FurE-W473A with wt FurE, (**Figure 5B**). These findings point to the idea that FurE molecules associate by oligomerization or partitioning into specific ER microdomains, early after their co-translational insertion into the ER, so that misfolded versions, such as FurE-Y484F or FurE-W473A, trap a fraction of wt FurE in aggregates incapable of ER exit.

### Truncation of TMS11-12 abolishes oligomerization or partitioning of FurE at ER-exit sites

**Figure 6:**
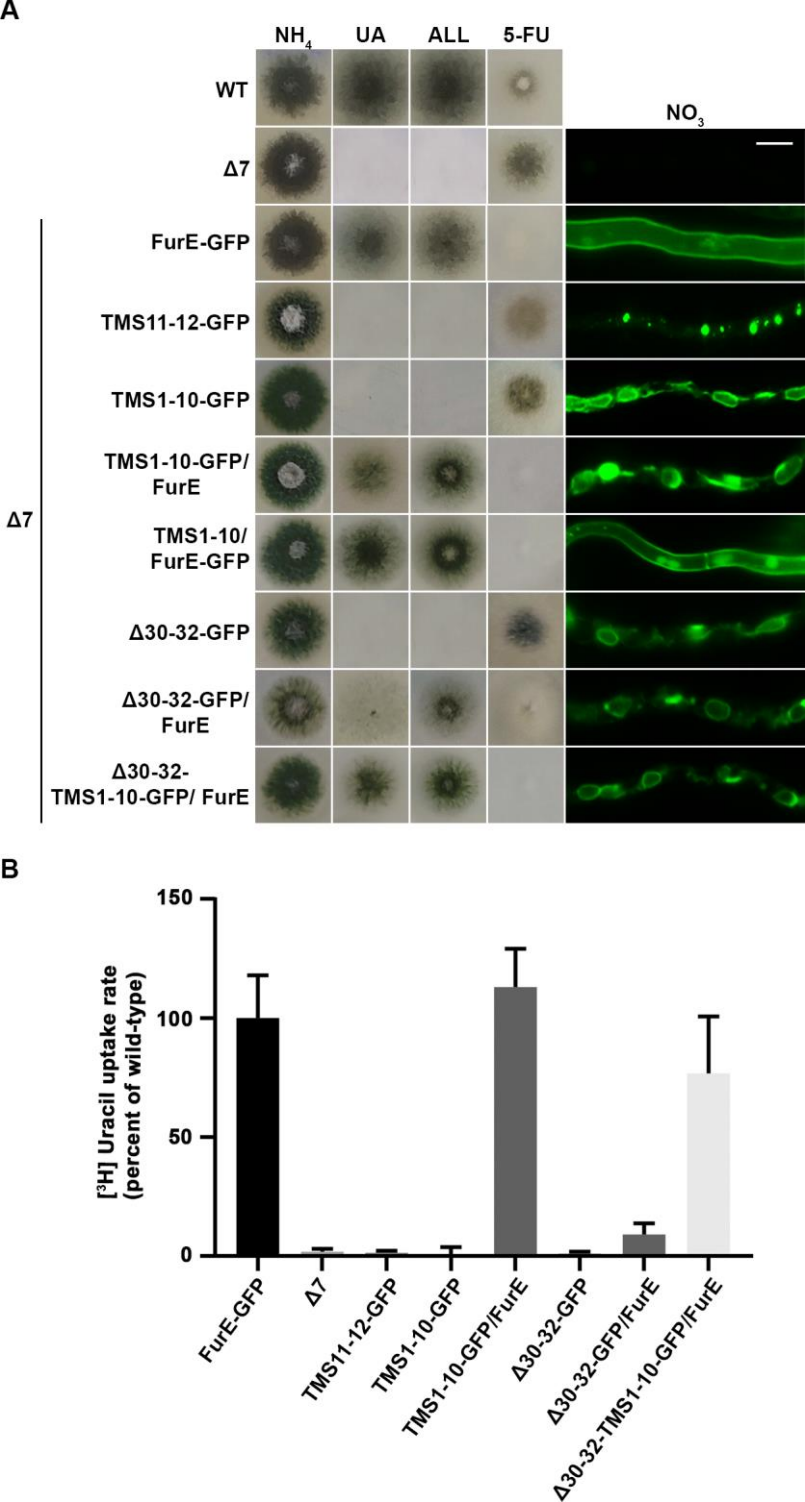
FIGURE 6: Truncated FurE versions containing the first 10 TMS are ER-retained and do not produce dominant negative effects when co-expressed with wild type FurE. **(A)** Growth tests and epifluorescence microscopy of controls, strains expressing truncated FurE versions con-sisting of TMS1-10 or TMS11-12, FurE molecules triple alanine substituted in positions 30–32 (Δ30–32) and strains co-expressing combinations of the above constructs with wild type FurE. In each strain one transporter version is GFP-tagged as noted in the strain name. Scale bar corresponds to 5 μM. **(B)** Radiolabeled uracil uptake rates, expressed as % of wt FurE rate, of strains co-expressing ER-retained FurE versions and wild-type FurE. For details see Materials and Methods.

To investigate the role of TMS11-12 in ER-exit and PM localization of FurE, we constructed and functionally analysed truncated versions of the transporter possessing either the core domains TMS1-10 (FurE-TMS1-10) or just the last two TMSs (FurE-TMS11-12). Both constructs were fused with GFP, and both were expressed via the *gpdA* promoter (for details see Materials and methods). First, we tested whether these two truncated versions translocate to the PM and whether FurE-TMS1-10 is functional. **Figure 6A** shows that FurE-TMS1-10 is totally trapped in the ER, and consequently could not confer FurE-dependent growth on allantoin or uric acid or sensitivity to 5FU, whereas FurE-TMS11-12 seems to be rapidly sorted in vacuole for degradation. We then co-expressed GFP-untagged FurE-TMS1-10 with FurE-TMS11-12-GFP, and tested whether the two parts of FurE are somehow functionally reconstituted or their co-expression promotes translocation to the PM. We showed that ‘split’ FurE could not be functionally or cellularly reconstituted (not shown). We also tried to employ Bifluorescence (BiF) assays using split YFP tags in all combinations (i.e., YFPn or YFPc N-terminally or C-terminally fused to the truncated parts of FurE), but still we did not obtain any evidence of reconstituted FurE parts (not shown). Subsequently, we co-expressed FurE-TMS1-10 with wt FurE and functionally analysed respective transformants. Results, summarized in **Figure 6A**, show no evidence for a dominant negative effect in respect to FurE function or localization, as seen when using non-truncated FurE versions.

To obtain further evidence for concentrative oligomerization or partitioning of FurE in ERes (ER exit sites) and the role of TMS11-12, we also employed a distinct version of FurE, not related to TMS11-12 that shows tight retention in the ER, and consequently no transport activity. This is a FurE mutant where N-terminal residues 30–32 (Leu-Asp-Ser) have been replaced by alanines [31] (named here FurE-Δ30-32). When full-length FurE-Δ30–32 was co-expressed with wt FurE, this led to synthetic phenotypes, basically a diminished transport capacity for uric acid and 5-fluorouracil (**Figure 6A**). However, when we used, in similar co-expression experiments, a truncated version of FurE-Δ30-32 lacking TMS11-12 (FurE-Δ30-32-TMS1-10), we ‘lost’ the dominant negative effect on growth (**Figure 6A**). In line with the above observations, direct uptake assays with radiolabeled uracil confirmed that FurE transport rates are significantly reduced in the strains co-expressing ER-retained FurE-Δ30-32 with wt FurE, while the uptake rate of strains co-expressing FurE-TMS1-10 or FurE-Δ30-32-TMS1-10 with wt FurE remained mostly unaffected (**Figure 6B**). Our findings strongly suggest that TMS11-12 are necessary for exit from the ER via their essential role in folding and oligomerization or partitioning of FurE in nascent ER exit sites.

## DISCUSSION

We showed that two specific aromatic residues in TMS12, namely Trp473 and Tyr484, are essential for ER-exit of FurE. In line with their functional importance, these residues are highly (Tyr484) or well (Trp473) conserved in FurE homologous proteins. Structural modelling and MD provided evidence that these residues, and especially Tyr484, might change their intramolecular topology relative to specific residues in other TMSs and aromatic residues at the end of TMS12, but also in respect to the lipid bilayer, during the transport cycle. This is a direct consequence of major topological changes that TMS12 undergoes during alteration from outward to inward conformation, as shown for Mhp1 [11, 13] and predicted by homology modelling in FurE here. More specifically, in the inward-facing conformation, TMS12 is shown to move away from the 5+5-fold core of the transporter and be directed towards annular lipids. Given the positioning of Trp473 in the middle of the bilayer and Tyr484 at the interphase of the transporter with lipids, the associated lack of proper ER-exit in the relative mutants suggests a structural defect in FurE folding due to modified intramolecular interactions and altered association with membrane lipids. In the case of Tyr484, we provided *in silico* evidence, supported by genetics, that this defect might be due to modified interactions of Tyr484 with two specific and highly conserved residues in TMS10 (Asp406) and TMS3 (Phe111), as well as, with downstream aromatic residues in TMS12 (mainly with Phe493).

Previous mutations in the N-terminal part of TMS10 (named TMS10**a**) have been shown to affect the function or specificity of FurE, albeit without affecting ER-exit (e.g., mutations in M389) [28]. Asp406, shown here to affect ER-exit, is located in the less flexible C-terminal part of TMS10 (named TMS10**b**). MD in Mhp1 has shown that the other half of TMS10 (TM10**a**) is dynamic gate able to occlude access to the major substrate binding site via local tilting in the middle of TMS10 [41]. It is thus probable that interaction of Tyr484 (TMS12) with Asp406 in TMS10**b** is a structural interaction necessary for proper FurE folding, but this interaction seems to also affect gating and transport via stabilization of the neighbouring dynamic movements of TMS10a. In conclusion, we have identified a putative dynamic network of structural interactions necessary for proper FurE folding and thus for ER-exit.

Our findings further show that specific TMS12 residues are involved principally in structural intramolecular interactions crucial for folding and proper ER-exit and localization to the PM, rather than being directly essential for substrate recognition and transport catalysis. Given the high conservation of residues involved in the network of interactions of TMS12, we predict that the ‘intramolecular chaperoning’ role TMS12 revealed herein might extent to all NCS1 or structurally similar APC-type transporters. In addition, the finding that the side-chain identity of most residues TMS12 and TMS11, except Trp473 and Tyr484, is little critical for ER-exit, further suggests that the packing of these helices with the 5+5 TMS core of the FurE transporter occurs via hydrophobic interactions of helical backbones, which are apparently strengthened by the specific interactions of involving Trp473 and Tyr484.

To our opinion, an original finding of this work is the discovery that co-expression of wt and ER-retained FurE leads to a synthetic dominant negative phenotype, which can be best explained by FurE oligomerization or partitioning in common ERes, and that this phenomenon is dependent on TMS11 and TMS12. We exclude the possibility that overexpression of ER-retained versions of FurE in some transformants causes a general defect in cargo trafficking as we did not observe any growth defects in the relative strains in all growth media tested, except those revealing a defect in FurE activity (e.g., growth on allantoin or uric acid or sensitivity to 5FU; See Supplementary Figure S5).

The observed inter-molecular interactions of FurE versions might be explained by tight oligomerization, as is the case in other transporters (e.g., the UapA uric acid-xanthine transporter of *A. nidulans*; [44]). However, we failed to obtain any evidence of FurE oligomerization at the ER or the PM, using a BiF approach or blue native gel electrophoresis (not shown). This lack of evidence for *in vivo* oligomerization is also true for other NCS1 transporters and several APC transporters. An alternative explanation of the observed dominant negative or positive phenotypes is that FurE molecules soon after their co-translational insertion into the ER membrane partition laterally in common microdomains, which associate with ERes. This has as a consequence the concentrative packaging of distinct FurE versions in common COPII vesicles. Concentrative ER-exit of membrane cargoes has been previously reported, suggesting that a multimeric sorting ‘code’ drives selectivity in cargo sorting [45].

Why are FurE mutants, such as those including mutations Y484F or W473A, unable to exit the ER when expressed by themselves? We believe these are partially misfolded FurE versions that do not provide the proper multimeric structure to partition properly in ERes. Misfolding of these mutants seems only partial, as at least in the case of Y484F and W473A, the relative FurE versions can exhibit extremely low, but still measurable minimal transport, in some transformants (not shown). This hypothesis suggests that properly folded and misfolded FurE molecules are capable of self-associating and partitioning in common microdomains, probably prior to sorting to ERes. In this case, there might be an intrinsic propensity of self-association or loose oligomerization of FurE molecules immediately after their biogenesis. Alternatively, an ER-associated adaptor protein might exist that specifically recognizes nascent FurE molecules and promote their association into a common microdomain or in ERes. Such cargo-specific ER adaptors for specific membrane cargoes exist, with the *Saccharomyces cerevisiae* Erv14 being the most extensively studied [46–48]. Mutants unable to exit the ER might then be unable to be recognized efficiently by this adaptor, or association with this adaptor is defective for proper exit from ERes.

An important finding related to the synthetic dominant negative phenotypes observed and the hypothesized association/oligomerization of FurE molecules in specific microdomains in the ER is the essential role of TMS11 and TMS12. We showed that these helices are essential for self-association of FurE molecules and/or partitioning to ERes. It is thus most reasonable to suggest that truncation of these helices leads to an unfolded FurE version, which unlike missense mutations in TMS3 (Phe111), TMS10 (Asp406), TMS12 (Trp473, Tyr484), or Δ30-32, cannot associate with co-expressed wt FurE molecules and thus fails to partition in ERes. This strengthens the notion that TMS11-12 function as an “intramolecular chaperone” essential for proper FurE folding, which in turn provides a structural code for FurE association and concentrative ER-exit the trafficking to the PM.

As already discussed in the Introduction, there is *in vitro* and *in vivo* evidence that several members of the APC superfamily oligomerize, and in some cases oligomerization was shown to depend on TMS11 and TMS12. For example, in AdiC, which has been shown to form homodimers *in vitro* and in possibly *in vivo*, the homodimer interface is formed by non-polar amino acids from TMS11 and TMS12, where residues of TMS11 from one monomer interdigitate with residues of TMS12 from the other monomer. Further interactions between the two monomers are mediated by the loops between TMS2 and TMS3, the cytoplasmic ends of TMS2 and TMS3, the cytoplasmic halves of TMS10, and the C-termini. The latter embrace neighbouring monomers [15]. However, given that monomers of AdiC have been reported to be transport active, the role of AdiC oligomerization remains unknown [15, 17]. Similarly, hSERT protomers have been shown to form oligomers via TMS11 and TMS12 [26]. Oligomeric states have also been proposed for rGAT1 and glycine transporters [24, 25]. Finally, the LeuT dimeric structure has also been shown to involve interactions of TMS9 and TMS12, and possibly TMS11 [18, 19]. In conclusion, findings presented in this work, concerning the essential role of TMS11 and TMS12 in FurE folding, self-association and/or concentrative sorting to nascent ERes, might well extend to other NCS1 and similar transporters of the APC superfamily.

## MATERIAL AND METHODS

### Media, strains and growth conditions

Standard complete (CM) and minimal media (MM) for *A. nidulans* growth were used. Media and supplemented auxotrophies were used at the concentrations given in http://www.fgsc.net [49]. Glucose 1% (w/v) was used as carbon source. 10 mM sodium nitrate (NO_3_) or 10 mM ammonium tartrate were used as standard nitrogen sources. Allantoin, uric acid and 5FU were used at the following final concentrations: 5FU at 100 μM; uric acid and allantoin 0.5 mM. All media and chemical reagents were obtained from Sigma-Aldrich (Life Science Chemilab SA, Hellas) or AppliChem (Bioline Scientific SA, Hellas). A Δ*furD::riboB* Δ*furA::riboB* Δ*fcyB::argB* Δ*azgA* Δ*uapA* Δ*uapC::AfpyrG* Δ*cntA::riboB pabaA1 pantoB100* mutant strain, named Δ7, was the recipient strain in transformations with plasmids carrying *furE* alleles based on complementation of the pantothenic acid auxotrophy *pantoB100* and/or the *pabaA1* auxotrophy [50]. A *pabaA1* (paraminobenzoic acid auxotrophy) is a wt control strain. *A. nidulans* protoplast isolation and transformation was performed as previously described [51]. Growth tests were performed at 37°C for 48 h, at pH 6.8.

### Standard molecular biology manipulations and plasmid construction

Genomic DNA extraction from *A. nidulans* was performed as described in FGSC (http://www.fgsc.net). Plasmids, prepared in *E. coli*, and DNA restriction or PCR fragments were purified from agarose 1% gels with the Nucleospin Plasmid Kit or Nucleospin ExtractII kit, according to the manufacturer's instructions (Macherey-Nagel, Lab Supplies Scientific SA, Hellas). Standard PCR reactions were performed using KAPATaq DNA polymerase (Kapa Biosystems). PCR products used for cloning, sequencing and re-introduction by transformation in *A. nidulans* were amplified by a high-fidelity KAPA HiFi HotStart Ready Mix (Kapa Biosystems) polymerase. DNA sequences were determined by VBC-Genomics (Vienna, Austria). Site directed mutagenesis was carried out according to the instructions accompanying the Quik-Change® Site-Directed Mutagenesis Kit (Agilent Technologies, Stratagene). The principal vector used for most *A. nidulans* mutants is a modified pGEM-T-easy vector carrying a version of the *gpdA* promoter, the *trpC* 3' termination region and the *panB* (and *pabaA* for co-transformations) selection marker. Mutations were constructed by oligonucleotide-directed mutagenesis or appropriate forward and reverse primers (see Supplementary Table S6).

### Protein Model Construction

The FurE modeled structure was constructed based on homology modeling using Prime 2018-4 (Schrödinger, LLC, New York, NY, 2018) on Maestro platform (Maestro, version 2018-4, Schrödinger, LLC, New York, NY, 2018). Mhp1 was used as query in the three conformations: Outward (2JLN), Occluded (4D1C), inward open (2X79), sharing with FurE a 35% similarity. The models shown here are presented with PyMOL 2.5 (https://pymol.org).

### Molecular Dynamics (MD)

Protein model construction and MD simulations are described in detail elsewhere [52]. In brief, homology models of FurE were constructed based on Mhp1 crystal structures 2JLN, 4D1B, 2X79. Each model was inserted into a lipid bilayer using the CHARMM-GUI tool and the resulting system was solvated using the TIP3P water model with final NaCl concentration of 150 mM. Calculations were conducted using GROMACS software, version 2019.2 and CHARMM36m force field [53, 54]. The protein orientation into the membrane was calculated using the PPM server (http://amber.manchester.ac.uk, [55]). The system was first minimized to obtain stable structures and then equilibrated for 20ns by gradually heating and releasing the restraints. The resulting equilibrated structures were then used as an initial condition for the production runs of 100ns at constant pressure of 1 atm and constant target temperature of 300K using Nose-Hoover thermostat and Parrinello-Rahman semi-isotropic pressure coupling.

### Transport assays

Kinetic analysis of wt and mutant FurE was measured by estimating uptake rates of [^3^H]-uracil uptake (40 Ci mmol^−1^, Moravek Biochemicals, CA, USA), as previously described [50]. In brief, [^3^H]-uracil uptake was assayed in *A. nidulans* conidiospores germinating for 4 h at 37°C, at 140 rpm, in liquid MM, pH 6.8. Initial velocities were measured on 10^7^ conidiospores/100 μL by incubation with concentration of 0.75 μM of [^3^H]-uracil at 37°C. The time points when the initial velocities (rates) are measured is 1 or 2 min. All transport assays were carried out at least in two independent experiments and the measurements in triplicate. Results were analysed in GraphPad Prism software.

### Epifluorescence microscopy

Samples for standard epifluorescence microscopy were prepared as previously described [56]. In brief, sterile 35 mm l-dishes with a glass bottom (Ibidi, Germany) containing liquid minimal media supplemented with NaNO_3_ and 1% glucose were inoculated from a spore solution and incubated for 16 h at 25°C. The images were obtained using an inverted Zeiss Axio Observer Z1 equipped with an Axio Cam HR R3 camera. Image processing and contrast adjustment were made using the ZEN 2012 software while further processing of the TIFF files was made using Adobe Photoshop CS3 software for brightness adjustment, rotation, alignment and annotation. The GFP-fluorescence intensity ratio (PM/cytosolic) was calculated using the ICY software [57]. The areas of the plasma membrane and the cytosol were manually highlighted and the intensity of GFP-fluorescence was measured. For nuclear staining, the DAPI dye was added to the growth medium in a final concentration of 0,002 mg/ml. The strains of interest were incubated with the dye for 20 minutes (25°C) and then washed with liquid minimal medium, before observation.

### Data Availability Statement

Strains and plasmids are available upon request. The authors affirm that all data necessary for confirming the conclusions of the article are present within the article, figures, and tables.

## SUPPLEMENTAL MATERIAL

Click here for supplemental data file.

All supplemental data for this article are available online at www.microbialcell.com/researcharticles/2023a-pyrris-microbial-cell/.

## Abbreviations:

5FU: – 5-fluorouracil,
APC: – amino acid-polyamine-oragnocation,
BiF: – bifluorescence,
MD: – molecular dynamics,
PM: – plasma membrane,
TMS: – transmembrane segment,
wt: – wild-type
