# Supplementary material for: The last two transmembrane helices in the APC-type FurE transporter act as an intramolecular chaperone essential for concentrative ER-exit
Source: Microb Cell. 2024 Jan 5;11:1–15. doi: 10.15698/mic2024.01.811 (PMC10788122; doi:10.15698/mic2024.01.811)
Supplement: Supplementary file 1 [file mic-11-001-s01.pdf]

The last two transmembrane helices in the APC-type FurE transporter act as an intramolecular chaperone essential for concentrative ER-exit

Yiannis Pyrris[1], Georgia F. Papadaki[1,#], Emmanuel Mikros[2] and George Diallinas[1,3,*]

[1]Department of Biology, National and Kapodistrian University of Athens, Panepistimioupolis, Athens, 15784, Greece.

[2]Department of Pharmacy, National and Kapodistrian University of Athens, Panepistimioupolis, Athens, 15771, Greece.

[3]Institute of Molecular Biology and Biotechnology, Foundation for Research and Technology, Heraklion, 70013, Greece.

Contact information: George Diallinas, diallina@biol.uoa.gr

Running title: Role of last two transmembrane helices in NCS1 transporter

Supplementary Material

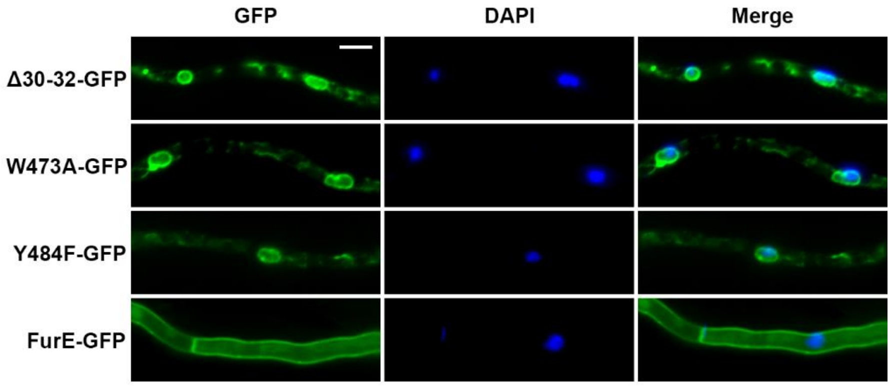

Supplementary figure S1. Trafficking-deficient, GFP-tagged, mutant FurE versions decorate characteristic perinuclear ER rings as well as cortical ER. Epifluorescence microscopy images of strains expressing GFP-tagged FurE versions after nuclear staining using the DAPI dye. The images from the GFP and DAPI channels as well as their merge are presented. For details see materials and methods.

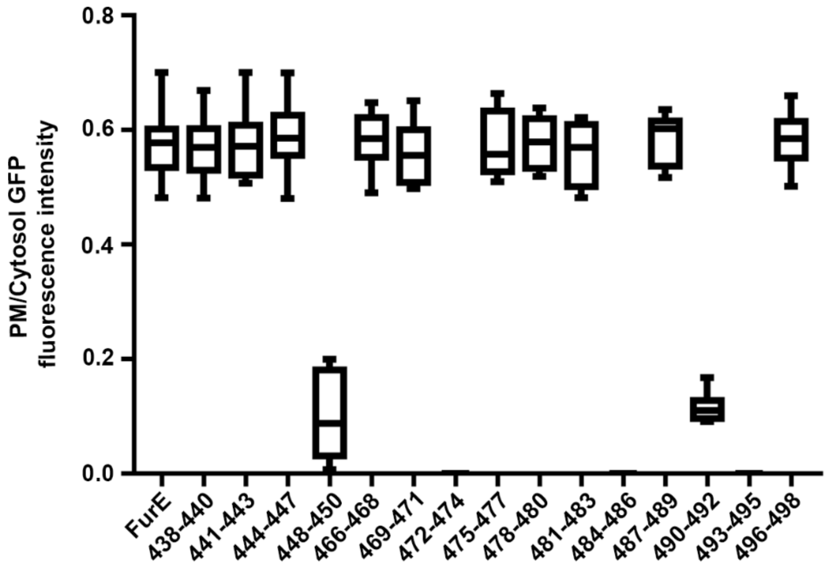

Supplementary figure S2. Most mutations in TMS11 and 12 do not severely affect the quantity of FurE in the PM. The boxplot represents the PM/Cytosol GFP fluorescence intensity of strains expressing different GFP-tagged FurE versions. The quantification was made using the ICY imaging software (see methods). The results correspond to 10 technical and 3 biological replicates.

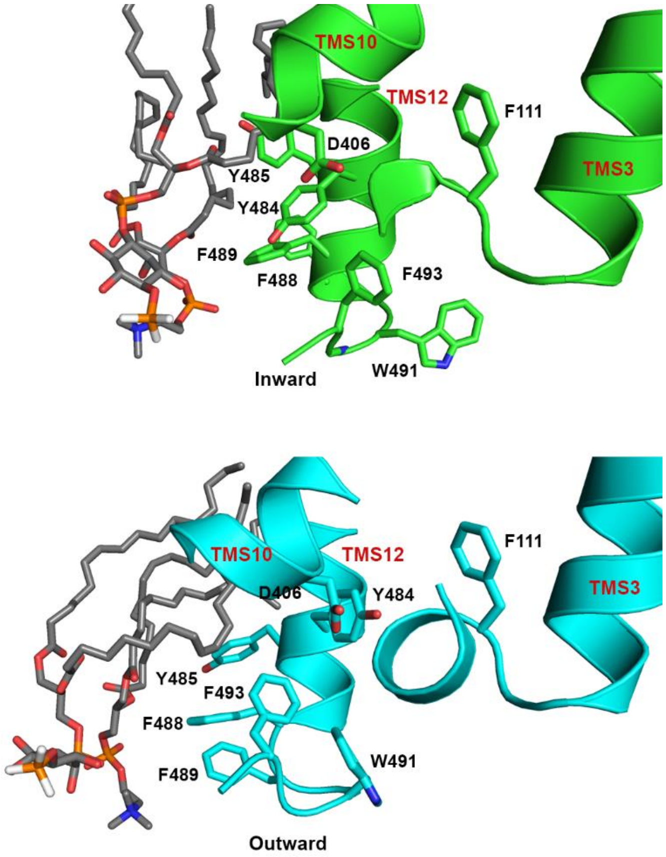

Supplementary figure S3. Y484 participates in an aromatic network that mediates interactions with annular lipids. Image was produced using PyMOL 2.5. For details on MD simulations see methods.

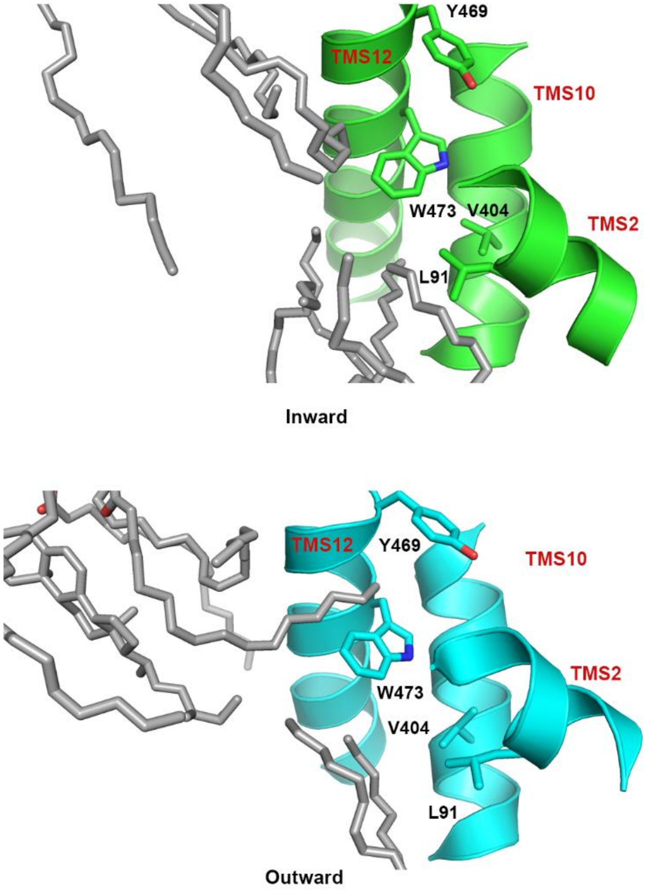

Supplementary figure S4. W473 directly interact with lipid tails in the middle of the bilayer.

Image was produced using PyMOL 2.5. For details on MD simulations see methods.

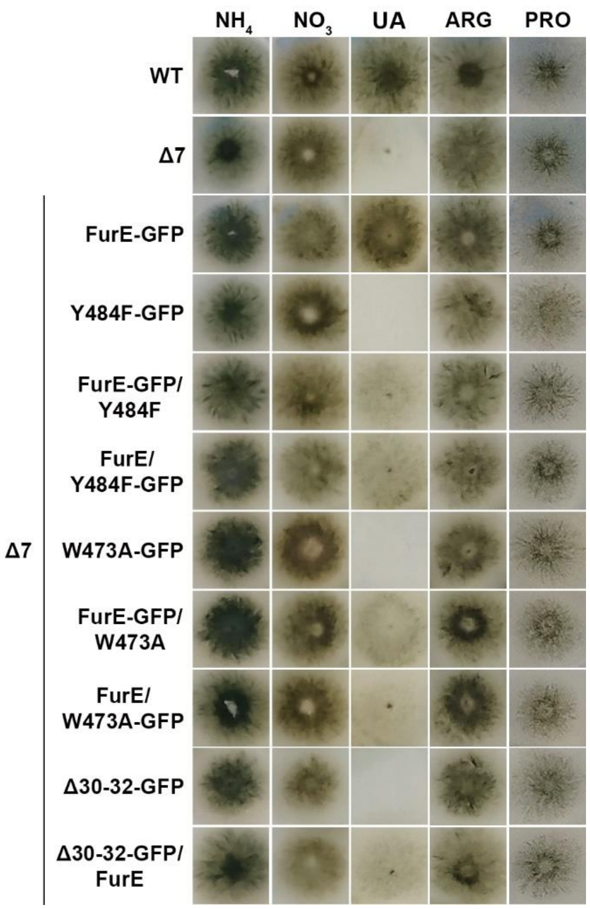

Supplementary figure S5. ER-retained FurE mutants do not impair the function of multiple other nitrogen compound transporters. Growth tests of control strains and strains expressing combinations of ER-retained mutant FurE versions and wild type FurE transporter. Growth tests were performed on MM supplemented with ammonium (NH_4), nitrate (NO_3), uric acid (UA), arginine (ARG) or proline (PRO) as nitrogen sources. The ER-retained mutants lead reduced transport capacity for uric acid (FurE substrate) and not for the other compounds.

Primer name	Oligonucleotide sequence
FurE d466-468 F	GCGTGCCGAAGGGAGCGGCGGCCGCGTACAGCTGCAGTTGGTTGG
FurE d466-468 R	CCAACCAACTGCAGCTGTACGCGGCCGCCGCTCCCTTCGGCACGC
FurE d469-471 F	GAAGGGAGCGAATTACTTGGCGGCCGCCAGTTGGTTGGTGAGCATTG
FurE d469-471 R	CAATGCTCACCAACCAACTGGCGGCCGCCAAGTAATTCGCTCCCTTC
FurE d472-474 F	CGAATTACTTGTACAGCTGCGCGGCCGCGGTGAGCATTGTTGTTTCTG
FurE d472-474 R	CAGAAACAACAATGCTCACCGCGGCCGCGCAGCTGTACAAGTAATTCG
FurE d475-477 F	GTACAGCTGCAGTTGGTTGGCGGCCGCTGTTGTTTCTGGGATGGTC
FurE d475-477 R	GACCATCCCAGAAACAACAGCGGCCGCCAACCAACTGCAGCTGTAC
FurE d478-480 F	CAGTTGGTTGGTGAGCATTGCGGCCGCTGGGATGGTCTATTACTTG
FurE d478-480 R	CAAGTAATAGACCATCCCAGCGGCCGCAATGCTCACCAACCAACTG
FurE d481-483 F	GGTGAGCATTGTTGTTTCTGCGGCCGCCTATTACTTGCTGTTTTTTG
FurE d481-483 R	CAAAAAACAGCAAGTAATAGGCGGCCGCAGAAACAACAATGCTCACC
FurE d484-486 F	GTTGTTTCTGGGATGGTCGCGGCCGCGCTGTTTTTTGTCTGGCCG
FurE d484-486 R	CGGCCAGACAAAAAACAGCGCGGCCGCGACCATCCCAGAAACAAC
FurE d487-489 F	CTGGGATGGTCTATTACTTGGCGGCCGCTGTCTGGCCGTTTGATG
FurE d487-489 R	CATCAAACGGCCAGACAGCGGCCGCCAAGTAATAGACCATCCCAG
FurE d490-492 F	CTATTACTTGCTGTTTTTTGCGGCCGCGTTTGATGTTGAAGAGAAAG
FurE d490-492 R	CTTTCTCTTCAACATCAAACGCGGCCGCAAAAAACAGCAAGTAATAG
FurE d493-495 F	GCTGTTTTTTGTCTGGCCGGCCGCCGCTGAAGAGAAAGTCATTGTGC
FurE d493-495 R	GCACAATGACTTTCTCTTCAGCGGCGGCCGGCCAGACAAAAAACAGC
FurE d496-498 F	CTGGCCGTTTGATGTTGCCGCTGCAGTCATTGTGCTTGAGGG
FurE d496-498 R	CCCTCAAGCACAATGACTGCAGCGGCAACATCAAACGGCCAG
FurE d438-440 F	GGTGTGAATATACGCGCCGCGGCCGCGTTTGTCTGCGGCATCG
FurE d438-440 R	CGATGCCGCAGACAAACGCGGCCGCGGCGCGTATATTCACACC
FurE d441-443 F	GAATATACGCGCCATGATCTCGGCGGCCGCCGGCATCGCGCCGAATC
FurE d441-443 R	GATTCGGCGCGATGCCGGCGGCCGCCGAGATCATGGCGCGTATATTC
FurE d444-447 F	CATGATCTCGTTTGTCTGCGCGGCCGCGGCGAATCTGCCTGGTTTG
FurE d444-447 R	CAAACCAGGCAGATTCGCCGCGGCCGCGCAGACAAACGAGATCATG
FurE d448-450 F	CTGCGGCATCGCGCCGGCGGCCGCTGGTTTGGCTGCGGTGAC
FurE d448-450 R	GTCACCGCAGCCAAACCAGCGGCCGCCGGCGCGATGCCGCAG
FurE W473A F	GAATTACTTGTACAGCTGCAGTGCGTTGGTGAGCATTGTTGTTTC
FurE W473A R	GAAACAACAATGCTCACCAACGCACTGCAGCTGTACAAGTAATTC
FurE W473F F	GAATTACTTGTACAGCTGCAGTTTCTTGGTGAGCATTGTTGTTTCTG
FurE W473F R	CAGAAACAACAATGCTCACCAAGAAACTGCAGCTGTACAAGTAATTC
FurE W473Y F	GAATTACTTGTACAGCTGCAGTTACTTGGTGAGCATTGTTGTTTCTG
FurE-W473Y R	CAGAAACAACAATGCTCACCAAGTAACTGCAGCTGTACAAGTAATTC
FurE L91A F	CGTTTGTATTCCGGCTATGGCCGATGGGTATGTATTGCCC
FurE-L91A R	GGGCAATACATACCCATCGGCCATAGCCGGAATACAAACG
FurE L91F F	CGTTTGTATTCCGGCTATGTTTGATGGGTATGTATTGCCC
FurE L91F R	GGGCAATACATACCCATCAAACATAGCCGGAATACAAACG
FurE V404A F	GTGCTATTGCTGGGGTGATTGCGGTTGATTATTGGGTTTGTCGG
FurE V404A R	CCGACAAACCCAATAATCAACCGCAATCACCCCAGCAATAGCAC
FurE Y484M F	CATTGTTGTTTCTGGGATGGTCATGTACTTGCTGTTTTTTGTC
FurE Y484M R	GACAAAAAACAGCAAGTACATGACCATCCCAGAAACAACAATG
FurE Y484F F	CATTGTTGTTTCTGGGATGGTCTTTTACTTGCTGTTTTTTGTC

FurE Y484F R	GACAAAAAACAGCAAGTAAAAGACCATCCCAGAAACAACAATG
FurE Y484S F	CATTGTTGTTTCTGGGATGGTCTCTTACTTGCTGTTTTTTGTC
FurE Y484S R	GACAAAAAACAGCAAGTAAGAGACCATCCCAGAAACAACAATG
FurE d30-32 F	CAAAGACCTCGACCCGGCCGCCGCCGACTCGCCCAAACGCAC
FurE d30-32 R	GTGCGTTTGGGCGAGTCGGCGGCGGCCGGGTCGAGGTCTTTG
FurE D406A F	GCTGGGGTGATTGTGGTTGCTTATTGGGTTTGTCGGGGGC
FurE D406A R	GCCCCCGACAAACCCAATAAGCAACCACAATCACCCCAGC
FurE F111A F	GTCTACACTCGAGCCAGCGCCGGTATGAAGGGGAGCTAC
FurE F111A R	GTAGCTCCCCTTCATACCGGCGCTGGCTCGAGTGTAGAC
FurE R123A F	GCTACTTCGCCGTCTTCGTTGCAGGGATTGTCGCTATTATCTGG
FurE R123A R	CCAGATAATAGCGACAATCCCTGCAACGAAGACGGCGAAGTAGC
FurE 11-12 NS speI F	GCGACTAGTATGGGTACGCACTACTTCACCAAAGG
FurE NotI R	GCGGCGGCCGCTGCAGAGACAGCCTCCTTC
FurE SpeI F	GCGACTAGTATGGGACTACGAGAAAGACTC
FurE 1-10 NS NotI R	CGCGCGGCCGCATGAGCTTCGTACAGTGAGCGC
FurE 1-10 NotI R	CGCGCGGCCGCCTAATGAGCTTCGTACAGTGAGCGC
FurE-F111Y-F	GTCTACACTCGAGCCAGCTACGGTATGAAGGGGAGCTAC
FurE-F111Y-R	GTAGCTCCCCTTCATACCGTAGCTGGCTCGAGTGTAGAC
FurE-D406E-F	GCTGGGGTGATTGTGGTTGAATATTGGGTTTGTCGGGGGCG
FurE-D406E-R	CGCCCCCGACAAACCCAATATTCAACCACAATCACCCCAGC

Supplementary Table S6. Primers used in this study. Primers for site directed mutagenesis contain the mutation on their names (e.g.Y484F). Primers used for constructing the truncated FurE versions (e.g., 1-10) contain restriction enzyme extensions as suggested by their names. Also the term “NS” stands for non-stop meaning that the stop codon has been omitted to enable GFP-fusions. The terms “F” and “R” mean forward and reverse. All primers are written as 5’-3’.

Protein	Uniprot accession number	Organism	Major substrate
FurE	Q5ATG4	*Aspergillus nidulans*	uric acid, allantoin, uracil
FurD	A6N844	*Aspergillus nidulans*	uracil, uric acid
Fur4	P05316	*Saccharomyces cerevisiae*	uracil
Dal4	Q04895	*Saccharomyces cerevisiae*	allantoin
Thi7	Q05998	*Saccharomyces cerevisiae*	thiamine
Nrt1	Q08485	*Saccharomyces cerevisiae*	nicotinamide riboside, thiamine
PLUTO	Q9LZD0	*Arabidopsis thaliana*	nucleobases
FurA	Q5BFM0	*Aspergillus nidulans*	allantoin
Mhp1	D6R8X8	*Microbacterium liquefaciens*	5-aryl-substituted hydantoins
FcyB	C8V329	*Aspergillus nidulans*	Adenine, hypoxanthine, cytosine, guanine
Fcy2	P17064	*Saccharomyces cerevisiae*	Adenine, hypoxanthine
Tpn1	P53099	*Saccharomyces cerevisiae*	pyridoxine

Supplementary Table S7. Uniprot accession numbers, taxonomy and major substrates of transporters aligned in Figure 1.
